# Activity of Selected Nucleoside Analogue ProTides against Zika Virus in Human Neural Stem Cells

**DOI:** 10.3390/v11040365

**Published:** 2019-04-20

**Authors:** Jean A. Bernatchez, Michael Coste, Sungjun Beck, Grace A. Wells, Lucas A. Luna, Alex E. Clark, Zhe Zhu, David Hecht, Jeremy N. Rich, Christal D. Sohl, Byron W. Purse, Jair L. Siqueira-Neto

**Affiliations:** 1 Skaggs School of Pharmacy and Pharmaceutical Sciences, University of California, San Diego, La Jolla, CA 92093, USA; jbernatchez@ucsd.edu (J.A.B.); sjbeck@ucsd.edu (S.B.); alexclark@ucsd.edu (A.E.C.); 2 Center for Discovery and Innovation in Parasitic Diseases, University of California, San Diego, La Jolla, CA 92093, USA; 3 Department of Chemistry and Biochemistry, San Diego State University, San Diego, CA 92182, USA; michael.coste1@gmail.com (M.C.); graceawells@gmail.com (G.A.W.); luluna@ad.ucsd.edu (L.A.L.); dhecht@swccd.edu (D.H.); 4 Department of Cellular and Molecular Medicine, University of California, San Diego, La Jolla, CA 92093, USA; 5 Sanford Consortium for Regenerative Medicine, La Jolla, CA 92093, USA; zhz504@ucsd.edu (Z.Z.); drjeremyrich@gmail.com (J.N.R.); 6 Department of Medicine, Division of Regenerative Medicine, School of Medicine, University of California, San Diego, La Jolla, CA 92093, USA; 7 Department of Chemistry, Southwestern College, Chula Vista, CA 91910, USA; 8 The Viral Information Institute, San Diego State University, San Diego, CA 92182, USA

**Keywords:** Zika virus, nucleoside analogues, antiviral agents, NS5, prodrugs, ProTides, neural stem cells, RNA-dependent RNA polymerase

## Abstract

Zika virus (ZIKV), an emerging flavivirus that causes neurodevelopmental impairment to fetuses and has been linked to Guillain-Barré syndrome continues to threaten global health due to the absence of targeted prophylaxis or treatment. Nucleoside analogues are good examples of efficient anti-viral inhibitors, and prodrug strategies using phosphate masking groups (ProTides) have been employed to improve the bioavailability of ribonucleoside analogues. Here, we synthesized and tested a small library of 13 ProTides against ZIKV in human neural stem cells. Strong activity was observed for 2′-*C*-methyluridine and 2′-*C*-ethynyluridine ProTides with an aryloxyl phosphoramidate masking group. Substitution of a 2-(methylthio) ethyl phosphoramidate for the aryloxyl phosphoramidate ProTide group of 2′-*C*-methyluridine completely abolished antiviral activity of the compound. The aryloxyl phosphoramidate ProTide of 2′-*C*-methyluridine outperformed the hepatitis C virus (HCV) drug sofosbuvir in suppression of viral titers and protection from cytopathic effect, while the former compound’s triphosphate active metabolite was better incorporated by purified ZIKV NS5 polymerase over time. These findings suggest both a nucleobase and ProTide group bias for the anti-ZIKV activity of nucleoside analogue ProTides in a disease-relevant cell model.

## 1. Introduction

The explosive spread of Zika virus (ZIKV) during the 2015–2016 epidemics in Latin America attracted worldwide attention to this previously neglected disease. The lack of effective vaccines or small molecules to prevent or treat this infection remains a cause for concern and emphasizes the urgent need for new therapeutic options [1]. ZIKV, an emerging flavivirus infection, causes several serious neurodevelopmental anomalies in the fetus, including microcephaly, congenital ZIKV syndrome (CZS), and even fetal demise [2]. While most cases of ZIKV infection are asymptomatic, reports of rash, conjunctivitis, pain in the muscles or joints, and fever have been recorded in clinical manifestations of the disease, and in rare cases, ZIKV infection has been linked to the neuroinflammatory Guillain-Barré syndrome [2].

Viral polymerases remain attractive drug targets for the development of selective antiviral therapies [3,4,5]. Generally, clinically-approved inhibitors that target these proteins fall into two broad classes [6,7]. The first class consists of nucleoside analogues that mimic the natural substrate of the enzyme. Upon analogue incorporation by the virally-encoded polymerase, DNA or RNA synthesis is abrogated by preventing further nucleotide incorporation (chain termination), thereby arresting viral replication. The second class is known as non-nucleoside inhibitors, which bind allosterically and arrest viral nucleic acid synthesis by distorting the polymerase active site geometry to interfere with nucleotide binding or nucleotide incorporation.

A common prodrug strategy used for antiviral ribonucleoside analogues involves the chemical synthesis of nucleoside analogue monophosphates with metabolically-removable masking groups [8,9,10]. These masking groups neutralize the negative charge of the phosphate on the nucleoside and allow for better membrane penetrance of these compounds. Chemical addition of the first phosphate group is crucial for improving intracellular levels of the active triphosphate metabolite form of ribonucleoside analogues. Metabolic addition of the first phosphate group is relatively slow for this class of compounds and represents a rate-limiting step for the triphosphorylation of ribonucleoside analogues to active drug molecules.

This “ProTide” strategy has been successfully deployed in the synthesis of bioactive ribonucleoside analogues [11,12], including the Food and Drug Administration-(FDA) approved hepatitis C virus (HCV) drug, sofosbuvir [13]. Sofosbuvir has recently been shown by multiple groups to also be active against ZIKV in vitro and in vivo [14,15,16,17], demonstrating that ribonucleoside analogue ProTides are an attractive avenue for the development of novel, selective antivirals against ZIKV.

Numerous nucleoside analogues have recently been explored as antiviral agents against ZIKV [18,19,20,21,22,23]. However, many of these compounds were tested in either cell-free systems or using cells that are not clinically relevant for the disease (such as Vero cells). Further, sofosbuvir is the only FDA approved inhibitor tested against ZIKV thus far in ProTide form. In addition, recent work by our group and others has suggested that sofosbuvir and ProTides in general have differential activity depending on the cell line used, which may be linked to the cell-specific metabolism of ProTides [17,24]. In this work, we chemically synthesized a library of 13 ProTides and tested them for activity against ZIKV in human neural stem cells, a disease-specific cell model of infection. Interestingly, the activity of 2′-*C*-modified aryloxyl phosphoramidate ProTides was strongly biased towards uridylate derivatives, with only modest activity observed for the 2′-*C*-modified adenylate and cytidylate aryloxyl phosphoramidate ProTides we tested. Changing the aryloxyl phosphoramidate masking group to a 2-(methylthio)ethyl phosphoramidate abolished the activity of the 2′-*C*-methyluridine ProTide, a representative hit from our library. In a head-to-head comparison between the 2′-*C*-methyluridine aryloxyl phosphoramidate ProTide and sofosbuvir, better suppression of viral titers was observed for the former compound. In addition, better protection from ZIKV-induced cytopathic effect was observed for the 2′-*C*-methyluridine aryloxyl phosphoramidate ProTide over sofosbuvir in a highly ZIKV-susceptible stem cell model of infection with strong phenotypic changes during ZIKV infection. These results represent the first broad study of nucleoside analogue ProTides against ZIKV.

## 2. Materials and Methods

### 2.1. Chemistry

**Scheme 1:**
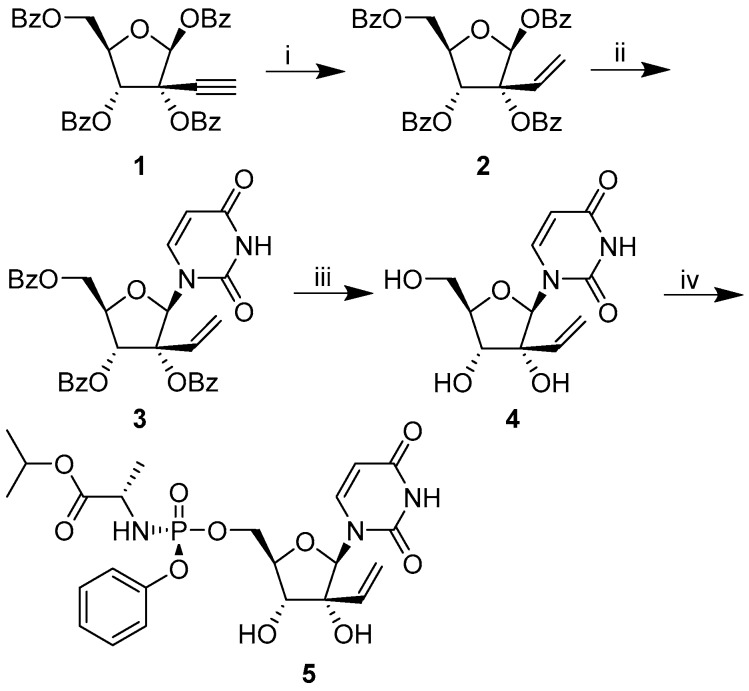
Synthesis of 2′-*C*-ethenyluridine ProTide. (i) H_2_ (g), Pd/BaSO_4_, quinoline, benzene, 78%; (ii) bovine serum albumin (BSA), uracil, SnCl_4_, ACN, 60 °C to 80 °C, 55%; (iii) NaOMe, MeOH, 0 °C to reflux, 94%; (iv) tBuMgCl, *N*-[(*S*)-(2,3,4,5,6-pentafluorophenoxy)phenoxyphosphynyl]-*L*-alanine 1-methylethyl ester, THF, −70 °C to room temperature (RT), 45%.

All nucleoside analogue ProTides, including McGuigan aryloxy phosphoramidite ProTides and 2-(methylthio)ethyl tryptamine ProTides, were synthesized using established phosphorus chemistry [25,26]. The 2′-*C*-ethenyluridine ProTide was synthesized using a known ethynyl ribose precursor **1**, which was obtained by following a previously reported synthetic route (Scheme 1) [27]. The ethenyl group of compound **2** was obtained by hydrogenation of the alkyne using 5% Pd/BaSO_4_ in (1:1) quinoline/benzene [28]. Ribosylation of uracil to compound **2** using *N*,*O*-bis(trimethylsilyl)acetamide (BSA) and SnCl_4_ in ACN afforded benzoyl-protected 2′-*C*-ethenyluridine **3**. The deprotection of compound **3** was performed using NaOMe in MeOH to afford 2′-*C*-ethenyluridine **4**, which was then treated with *t*BuMgCl in THF followed by *N*-[(*S*)-(2,3,4,5,6-pentafluorophenoxy)phenoxyphosphinyl]-l-alanine 1-methylethyl ester and to afford 2′-*C*-ethenyluridine aryloxy phosphoramidate **5**.

#### 2.1.1. General Synthetic Experimental

All reagents, chemicals, and nucleoside analogues precursors were purchased from Acros Organics, Carbosynth, Chem-Impex International, AK Scientific, and Fisher Chemical at ACS reagent grade or higher purity and used as received. The (3R,4R,5R)-5-(benzoyloxy)methyl)-3-ethynyltetrahydrofuran-2,3,4-triyl tribenzoate **1** was synthesized by following a previously reported procedure [27]. Synthetic details for aryloxy phosphoramidate ProTides other than for 2′-*C*-ethynyluridine and 2′-*C*-ethenyluridine were published previously by our group [24]. All reactions were carried out in either an oven-dried round bottom flask or a Schlenk tube under a nitrogen atmosphere using commercially available anhydrous solvents and monitored by thin-layer chromatography with detection by UV light. The ^1^H NMR and the ^31^P NMR spectra were acquired on a Varian 400-MHz spectrometer and recorded at 298 K. Chemical shifts were referenced to the residual protio solvent peak and are given in parts per million (ppm). Splitting patterns are denoted as s (singlet), d (doublet), dd (doublet of doublet), dq (doublet of quartet), ddd (doublet of doublet of doublet), ddt (doublet of doublet of triplet), t (triplet), td (triplet of doublet), tdd (triplet of doublet of doublet), q (quartet), and m (multiplet).

##### 2′-*C*-Ethynyluridine Aryloxy Phosphoramidate 

To a stirred suspension of 2′-*C*-ethynyluridine (0.09 mmol, dried under vacuum at 50 °C overnight) in dry THF (1 mL) was added a 2.0 M solution of tert-butyl magnesium chloride in THF (96 μL, 0.19 mmol). The mixture was stirred at 0 °C for 30 min and then allowed to warm to room temperature and stirred for an additional 30 min. The reaction mixture was then cooled to 0 °C and *N*-[(*S*)-(2,3,4,5,6-pentafluorophenoxy)phenoxyphosphinyl]-L-alanine 1-methylethyl ester (46 mg, 0.10 mmol) was added. The reaction mixture was stirred for 18 h as the temperature was allowed to warm to room temperature. The solvent was removed by rotary evaporation. The reaction mixture was purified first using flash chromatography (a gradient from 0 to 30% methanol in dichloromethane) and then using preparative, normal-phase HPLC (10 to 40% MeOH in dichloromethane gradient) to afford 25.5 mg of the product (54.3%) in ≥ 95% purity. ^1^H NMR (400 MHz, CD_3_OD): δ= 7.65 (d, 1H, *J* = 8.1 Hz), 7.40–7.34 (m, 2H), 7.27 (dq, 2H, *J* = 7.7, 1.2 Hz), 7.20 (ddd, 1H, *J* = 8.2, 7.1, 1.1 Hz), 6.04 (s, 1H), 5.61 (d, 1H, *J* = 8.1 Hz), 4.97 (m, 1H), 4.49 (ddd, 1H, *J* = 11.8, 6.0, 2.1 Hz), 4.36 (ddd, 1H, *J* = 11.8, 6.1, 3.7 Hz), 4.16 (d, 1H, *J* = 9.1 Hz), 4.08 (ddt, 1H, *J* = 9.0, 3.9, 2.1 Hz), 3.92 (dq, 1H, *J* = 9.9, 7.1 Hz), 3.08 (s, 1H), 1.35 (dd, 3H, *J* = 7.2, 1.0 Hz), 1.22 (dd, 6H, *J* = 6.3, 1.8 Hz). ^31^P NMR (162 MHz, CD_3_OD) δ= 3.78.

##### 5-((Benzoyloxy)methyl)-3-vinyltetrahydrofuran-2,3,4-triyl tribenzoate **2**

To a stirring solution of **1** (208.7 mg, 0.362 mmol, 1 equivalent) in benzene (2 mL) and ethanol (2 mL) under H_2_ (g), we added 5% palladium on barium sulfate (20.8 mg, 10 wt%) followed by quinolone (22 µL) and stirred at room temperature for 2 h. The mixture was diluted in ethyl acetate, washed 3 times with water, and dried over anhydrous sodium sulfate. The reaction mixture was purified using flash chromatography (0 to 30% ethyl acetate in hexane gradient) to afford purified product; (162.7 mg, 0.281 mmol, 77.7%) 1H NMR (400 MHz, CDCl_3_): δ = 8.23–8.09 (m, 4H), 8.07–8.03 (m, 2H), 7.92–7.88 (m, 2H), 7.69–7.39 (m, 10H), 7.18–7.12 (m, 2H), 6.46 (dd, 1H, *J* = 17.6, 11.2 Hz), 6.25 (d, 1H, *J* = 8.3 Hz), 4.54 (dd, 2H, *J* = 12.2, 4.8 Hz), 4.81 (ddd, 2H, *J* = 8.4, 4.7, 3.9 Hz), 4.73 (dd, 1H, *J* = 12.2, 3.9 Hz), 4.54 (dd, 1H, *J* = 12.2, 4.8 Hz)

##### 5-((Benzoyloxy)Methyl)-2-(2,4-Dioxo-3,4-Hihydropyrimidin-1(2H)-yl)-3-Vinyltetrahydrofuran-3,4-diyl Benzoate **3**

Uracil (63.0 mg, 0.562 mmol, 2 equivalent) and **2** (162.7 mg, 0.281 mmol, 1 equivalent) were dried under high vacuum in separate round bottom flasks for 2 h. Under N_2_ (g) and stirring, we added dry acetonitrile (2 mL) to uracil followed by the addition of bis(trimethylsilyl)acetamide (550.1 µL, 2.250 mmol, 8 equivalent). The reaction mixture was refluxed at 80 °C for 1 h then cooled to 0 °C. Then, compound 2 in dry acetonitrile (2 mL) was added to the reaction mixture followed by tin (IV) chloride (229.9 µL, 1.968 mmol, 7 equivalent) and heated to 60 °C for 3 h. The reaction mixture was poured into a separatory funnel containing ice cold water, extracted 3 times with ethyl acetate, and the combined organic layer was dried over anhydrous sodium sulfate. The reaction mixture was purified using flash chromatography (0 to 100% ethyl acetate in hexane gradient) to afford purified product; (89.4 mg, 0.153 mmol, 54.6%) 1H NMR (400 MHz, CDCl_3_): δ= 9.22 (s, 1H), 8.09 (m, 4H), 7.86–7.82 (m, 2H), 7.63–7.56 (m, 2H), 7.63–7.56 (m, 6H), 7.29–7.21 (m, 2H), 6.65 (s, 1H), 6.12 (dd, 1H, *J* = 17.5, 11.1 Hz), 6.04 (d, 1H, *J* = 5.2 Hz), 5.64 (dd, 1H, *J* = 8.2, 2.1 Hz), 5.46–5.40 (dd, 2H), 4.94 (dd, 1H, *J* = 12.3, 3.2 Hz), 4.81 (dd, 1H, *J* = 12.3, 5.7 Hz), 4.66 (td, 1H, *J* = 5.5, 3.2 Hz)

##### 1-(3,4-dihydroxy-5-(hydromethyl)-3-vinyltetrahydrofuran2-yl)pyrimidine-2,4(1H,3H)-dione **4**

Compound **3** (90.5 g, 0.155 mmol, 1 equivalent) was dried overnight on high vacuum. Under N_2_ (g), methanol (1.5 mL) was added, then the reaction mixture was cooled to 0 °C followed by the dropwise addition of sodium methoxide (86.5 µL, 1.553 mmol, 10 equivalent). The reaction mixture was raised to room temperature and stirred for 1.5 h. The reaction mixture was cooled to 0 °C followed by the addition of formic acid until pH = 4. The reaction mixture was dried in vacuo then purified using flash chromatography (0 to 40% methanol in dicholoromethane gradient) to afford a purified product; (30.2 mg, 0.146 mmol, 93.8%) 1H NMR (400 MHz, CDCl_3_): δ= 8.13 (d, 1H, *J* = 8.1 Hz), 5.95 (s, 1H), 5.74–5.65 (m, 2H), 5.44 (dd, 1H, *J* = 17.3, 1.3 Hz), 5.26 (dd, 1H, *J* = 10.8, 1.3 Hz), 4.22 (d, 1H, *J* = 9.2 Hz), 4.03–3.97 (m, 2H), 3.84–3.79 (m, 1H)

##### isopropyl(((5-(2,4-dioxo-3,4-dihydropyridimin-1(2H)-yl)-3,4-dihydroxy-3-inyltetrahydrofuran-2-yl)methoxy)(phenoxy)phosphoryl)-L-alaninate **5**

To a stirring solution of **4** (36.4 mg, 0.176 mmol, 1 equivalent) in dry tetrahydrofuran (1 mL) at 0 °C was added tert-butyl magnesium chloride (184.5 µL, 0.369 mmol, 2.1 equivalent). The reaction mixture was raised to room temperature and allowed to react for 30 min. The reaction mixture was cooled to 0 °C, then *N*-[(*S*)-(2,3,4,5,6-pentafluorophenoxy)phenoxyphosphinyl]-L-alanine 1-methylethyl ester (95.5 mg, 0.211 mmol, 1.2 equivalent) was added and gradually warmed to room temperature overnight. The reaction mixture was purified using flash chromatography (0 to 30% methanol [MeOH] in dichloromethane gradient) to afford the purified product; (42.5 mg, 0.0788 mmol, 44.8%) 1H NMR (400 MHz, CD_3_OD): δ= 7.76 (d, 1H, *J* = 8.1 Hz), 7.38 (dd, 2H, *J* = 8.6, 7.2 Hz), 7.31–7.25 (m, 2H), 7.25–7.16 (m, 2H), 5.94 (s, 1H), 5.68 (dd, 1H, *J* = 17.3, 10.8 Hz), 5.60 (d, 1H, *J* = 8.1 Hz), 5.48 (d, 1H, *J* = 1.4 Hz), 5.44 (d, 1, *J* = 1.4 Hz), 5.27 (dd, 1H, *J* = 10.8, 1.4 Hz), 4.96 (1H, m), 4.58–4.49 (m, 1H), 4.45–4.38 (m, 1H), 4.17 (s, 2H), 4.00–3.87 (m, 1H), 1.38 (dd, 3H, *J* = 7.1, 1.0 Hz), 1.21 (d, 6H, *J* = 6.3 Hz). ^31^P NMR (162 MHz, CD_3_OD) δ = 3.78.

##### (5-(2,4-dioxo-3,4-dihydropyrimidin-1(2H)-yl)-3,4-dihydroxy-4-methyltetrahydrofuran-2-yl)methyl (2-(methylthio)ethyl) (2-(1H-indol-3-yl)ethyl)phosphoramidate (2′-*C*-methyluridine methylthioethyl tryptamine ProTide)

The 2′-*C*-methyluridine (45.3 mg, 0.1790 mmol, 1 equivalent) and the triethylammonium 2-(methylthio)ethyl phosphonate (92.1 mg, 0.3579 mmol, 1.5 equivalent) were added to an oven-dried schlenk tube and dried overnight on the high vacuum. To the flask under N_2_ (g) was added dry pyridine (2 mL) followed by the dropwise addition of trimethylacetyl chloride (60.6 µL, 0.4923 mmol, 2.75 equivalent), and the reaction was stirred for 3 h at room temperature. The reaction was quenched by saturated sodium bicarbonate and extracted 3 times with dicholormethane. The organic layer was dried over anhydrous sodium sulfate and concentrated in vacuo at room temperature and dried for 2 h on high vacuum. The dried residue was dissolved in pyridine (2 mL) under N_2_ (g). To the reaction mixture was simultaneously added trimethylamine (49.9 µL, 0.3579 mmol, 2 equivalent), tryptamine (114.7 mg, 0.7158 mmol, 4 equivalent, dissolved in dry pyridine 2 mL), and carbon tetrachloride (26.0 µL, 0.2685 mmol, 1.5 equivalent) and stirred for 25 min at room temperature. The reaction mixture was diluted with methanol and purified using flash chromatography (0 to 30% methanol in dichloromethane gradient) to afford a purified product as a mixture of two epimers at phosphorus; (8.0 mg, 0.01443 mmol, 7.3%) ^1^H NMR (400 MHz, CD_3_OD): δ= 7.72 (dd, 1H, *J* = 21.4, 8.2 Hz), 7.55–7.50 (m, 1H), 7.33–7.29 (m, 1H), 7.06 (t, 2H, *J* = 7.3 Hz), 6.97 (tdd, 1H, *J* = 7.5, 4.6, 2.4 Hz), 5.95 (d, 1H, *J* = 5.2 Hz), 5.64 (dd, 1H, *J* = 8.1, 3.8 Hz), 4.37–4.29 (m, 1H), 4.17 (ddd, 1H, *J* = 11.7, 5.0, 3.1 Hz), 4.04 (dt, 1H, *J* = 10.4, 6.8 Hz), 4.00–3.90 (m, 1H), 3.77 (dd, 1H, *J* = 12.4, 9.3 Hz), 3.21 (td, 2H, *J* = 10.9, 5.6 Hz), 2.96 (dt, 2H, *J* = 7.3, 4.4 Hz), 2.66 (td, 2H, *J* = 6.7, 2.3 Hz), 2.07 (d, 3H, *J* = 1.4 Hz), 1.13 (s, 3H). ^31^P NMR (162 MHz, CD_3_OD) δ= 10.52 and 10.41.

### 2.2. Cell Culture

Human fetal NSCs were purchased from Clontech (human neural cortex; catalog number Y40050). Maintenance of hNSCs was performed in Neurobasal medium without phenol red (Thermo Fisher, Waltham, MA, USA) and in the presence of B-27 supplement (1:100; catalog number 12587010; Thermo Fisher), N-2 supplement (1:200; catalog number 17502-048; Invitrogen, San Diego, CA, USA), 20 ng/mL fibroblast growth factor (catalog number 4114-TC-01M; R&D Systems, Minneapolis, MN, USA, 20 ng/mL epidermal growth factor (catalog number 236-EG-01M; R&D Systems), GlutaMAX (catalog number 35050061; Thermo Fisher), and sodium pyruvate. Cells were prepared in 6-well plates precoated with laminin (10 mg/mL; catalog number L2020; Sigma-Aldrich, St. Louis, MO, USA) and were grown to near confluence (80 to 90%) prior to passage. For passaging, cells were rinsed gently with phosphate-buffered saline (PBS) (without calcium and magnesium) and then treated with TryPLE for 5 min at 37 °C for cell detachment (catalog number 12563029; Thermo Fisher). The GSC 387 cell line was prepared and maintained similarly to what has been previously described using the media and detachment conditions used for hNSCs described above [29]. Vero cells (ATCC CCL-81) were purchased from ATCC and cultured in Dulbecco’s Modified Eagle Medium (DMEM) (Gibco, Waltham, MA, USA) with high glucose (4.5 g/L), 10% fetal calf serum (FCS) (Sigma), and 1% Penicillin-Streptomycin (PS) (Sigma-Aldrich, St. Louis, MO, USA) at 37 °C and 5% CO_2_.

### 2.3. Viruses

Viral strains used in this study were provided through BEI Resources, NIAID, NIH: Zika virus H/PAN/2016/BEI-259634, NR-50210 (GenBank accession number KX198135), Zika virus PRVABC59/Human/2015/Puerto Rico, NR-50240 (GenBank accession number KU501215).

Vero cells were used to expand viral cultures for 2 to 3 serial passages in order to amplify the titers. Centrifugation of infected cell supernatants was performed to remove cell debris, and then viral stocks were concentrated through use of a sucrose cushion. Neural maintenance medium base [50% DMEM–F-12 medium–GlutaMAX, 50% Neurobasal-A medium, 1× N-2 supplement, 1× B-27 supplement (Life Technologies)] supplemented with 1% DMSO (Sigma) and 5% fetal bovine serum (FBS) (Gibco) was used to resuspend concentrated viral stocks, and aliquots were stored at −80 °C. Plaque assay on Vero cells was used to determine viral titers, which were greater than or equal to 2 × 10^8^ PFU/mL.

### 2.4. CellTiter-Glo Activity Assay

NSCs (5000 cells/well) were seeded into 1536-well black, clear bottom plates containing 20-point, 2-fold serial dilutions of ProTides or DMSO vehicle controls and were either mock-infected, infected with ZIKV H/PAN/2016/BEI-259634 at the multiplicity of infection (MOI) of 10, or infected with ZIKV PRVABC59/Human/2015/Puerto Rico at MOI of 10. Cell viability was measured using the CellTiter-Glo assay (Promega, Madison, WI, USA) 72 h post-infection. An EnVision plate reader (PerkinElmer) was used for readouts of luminescence intensity. All data were normalized to DMSO vehicle controls and were expressed as % relative luminescence intensity. Normalized activity and toxicity data were plotted against ProTide concentration and fit to a sigmoidal dose-response curve with variable slope using Prism 6 (GraphPad Software) to generate EC_50_ and CC_50_ curves.

### 2.5. Bright-Field Microscopy

GSC 387 cells (5000 cells/well) were seeded into 1536-well black, clear bottom plates with wells containing either 10 or 30 µM sofosbuvir, 10 or 30 µM 2′-*C*-methyluridine aryloxyl phosphoramidate ProTide, or DMSO vehicle controls, and were either mock-infected or infected with ZIKV H/PAN/2016/BEI-259634 at MOI of 10. Cell foci images were acquired 72 h post-infection in bright-field using an ImageXpress Micro automated microscope (Molecular Devices, San Jose, CA, USA).

### 2.6. Plaque Assay

Vero cells were used for the titering experiments for drug-treated and vehicle-treated hNSC cultures infected with ZIKV H/PAN/2016/BEI-259634. Vero cells were seeded at a density of 30,000 cells per well in 96-well plates and incubated in 5% CO_2_ at 37 °C in high-glucose DMEM with 1% FBS and 1% penicillin/streptomycin for 24 h before the addition of the viral inoculum from ZIKV-infected hNSCs. Inocula were collected from ZIKV H/PAN/2016/BEI-259634-infected NSCs (MOI 0.1) in the presence of 10 μM or 30 μM 2′-*C*-methyluridine aryloxyl phosphoramidate ProTide, 10 μM or 30 μM sofosbuvir, mock-infected cultures, infected cultures without compound treatment, and infected cultures with the DMSO vehicle at 48 h post-infection. Collected inocula were diluted 10-fold in high-glucose DMEM containing 1% FBS and 1% penicillin-streptomycin in 96-well plates. These inocula were added to the pre-prepared Vero cells for 1 h in a volume of 100 µL and at a temperature of 37 °C, covered with an agarose overlay, and lastly incubated for 72 h at 37 °C and 5% CO_2_. Fixation with 4% formaldehyde (final concentration) for 24 h was then performed for the samples. Following this, the overlay was removed, and crystal violet staining was performed to count the ZIKV plaques.

### 2.7. ZIKV RdRP Expression and Purification

The RdRP domain of ZIKV NS5 (amino acids 2772-3423 of accession number AMB18850) was fused to an N-terminal 6×-histidine tag containing a TEV cleavage site, and the cDNA was codon-optimized and synthesized by IDT (San Diego, CA, USA). This construct was then inserted into a pET-17b vector (Invitrogen) and heterologously expressed in the Rosetta strain of *Escherichia coli*. Cultures transfected with the RdRP construct were grown at 37 °C in LB media containing 1% ethanol and 100 µg/mL amplicillin until an OD_600_ of 0.6–0.7 was reached. Protein expression was then induced with 100 µM IPTG prior to an additional 5 h incubation at 20 °C and lower speed shaking (130 RPM). Cells were then harvested by centrifugation at 9000 RPM for 30 min at 4 °C, and pellets were stored at −80 °C. For purification, pellets were resuspended in lysis buffer (50 mM HEPES at pH 7.5, 20 mM NaCl, 20% glycerol, 0.1% octyl-glucoside, 5 mM β-mercaptoethanol) supplemented with a protease inhibitor tablet (Thermo Fisher). Cells were lysed using a microfluidizer (Microfluidics International Corporation, Newton, MA, USA) and clarified via centrifugation at 12,000 RPM for 30 min at 4 °C. The supernatant was loaded onto TALON cobalt affinity beads (Clontech, Mountain View, CA, USA) at 4 °C. The unbound protein was washed with 50 mL each of the following buffers: wash buffer (50 mM HEPES at pH 7.5, 5 mM β-mercaptoethanol) containing 100 mM NaCl and 10 mM imidazole, wash buffer containing 500 mM NaCl and 20 mM imidazole, and finally wash buffer containing 200 mM NaCl and 30 mM imidazole. The protein was eluted in wash buffer containing 200 mM NaCl and a linear imidazole gradient of 30–300 mM. Protein fractions were pooled and dialyzed overnight against 50 mM HEPES at pH 7.0, 100 mM NaCl, 10% glycerol, 0.01% CHAPS, and 5 mM dithiothreitol at 4 °C. The protein was concentrated to ≥6 µM and stored in single-use aliquots at −80 °C.

### 2.8. Single Nucleotide Incorporation Assay

To prepare the double-stranded RNA substrate, primer (5′-AGUUGUUGAUC3′) was ^32^P-labeled at the 5′ position upon incubation with [γ-^32^P] ATP (PerkinElmer, Waltham, MA, USA) and T4 polynucleotide kinase (NEB, Ipswich, MA, USA) at 37 °C for 30 min. Excess [γ-^32^P] ATP was removed with a Bio-Spin 6 column (Bio-Rad, Hercules, CA, USA). After heat inactivation, the 5′-^32^P labeled primer was combined with an excess template (5′-AUUCACUCAGAUCAACAACU-3′). The primer and the template were annealed via step incubation at 95 °C (5 min), 55 °C (15 min), and 37 °C (10 min) to generate the primer/template substrate (P/T substrate), where the next correct nucleotide to be incorporated was UTP.

Single nucleotide incororation assays were modeled from previous work [19]. Briefly, ZIKV RdRP (1 µM) and P/T substrate (200 nM) were pre-incubated at 37 °C for 20 min. Reactions were initiated at 37 °C with the addition of 150 µM UTP or analog in 50 mM HEPES pH 7.5, 15% glycerol, 15 mM NaCl, 7.5 mM MnCl_2_, and 10 mM dithiothreitol. At specified time points, reaction aliquots were quenched with ethylenediamine tetra-acetic acid (EDTA) to a final concentration of 0.3 M.

Prior to loading equi-volume samples on a 20% polyacrylamide, 8 M urea, TBE (89 mM Tris-Borate, 2 mM EDTA) denaturing gel, the quenched samples were combined with dye (formamide, 0.01% xylene cyanol, 0.01% bromophenol blue) and 1 µL of 5 µM D10 (5′-AGTTGTTGAT-3′) trap, followed by incubation at 95 °C for 5 min. The trap was used to bind to RNA template to reduce gel smearing [30]. Oligos were separated via electrophoresis at a maximum of 1900 V and 75 W for 3–6 h. Phosphorimaging (FLA 9500 imager, GE, Marlborough, MA, USA) was used to visualize the bands, and bands were quantified using ImageQuant software (GE). Plots of percent incorporation versus time were fit to a single exponential equation using Prism (Graphpad Software, San Diego, CA, USA) to compare relative incorporation. While single-turnover conditions were used, non-physiologically relevant rates were obtained, as incorporation occurred during a steady-state time scale. This has been a common issue in the field when measuring ZIKV RdRP incorporation [19,20,31] due to measuring incorporation into a double-stranded template instead of physiologically preferred de novo RNA replication (an issue seen in HCV incorporation assays [32]), and perhaps the presence of purification tags [33]. However, since here we were interested in comparing relative incorporation efficiency rather than obtaining rate constants, we and others [19,31] have found this experimental set-up ideal and better suited to scaling up the number of analogues to be assessed.

### 2.9. Structural Alignment

The following model and structures were aligned to HCV RdRP complexed with RNA, Mn^2+^, and sofosbuvir-DP (PDB 4WTD [34]) using Pymol [35]: 1) a model of ZIKV RdRP in complex with RNA, Mn^2+^ ions, and an incoming ADP modified to an ATP (gc-o3 [36]) generated from ZIKV RdRP stucture PDB 5TFR [37] (160 atoms aligned, RMS = 1.650); 2) a crystal structure of apo ZIKV RdRP (PDB 5WZ3 [38]) (154 atoms aligned, RMS = 2.860); and 3) a crystal structure of apo HCV RdRP (PDB 3MWV [39]) (530 atoms aligned, RMS = 0.874).

## 3. Results

### 3.1. ProTide Library Design

**Figure 1:**
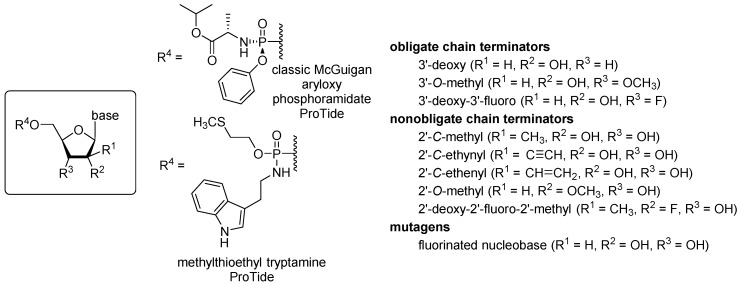
Design and structure of nucleotide analogue inhibitors tested against Zika virus (ZIKV) in this study.

To investigate the activity of a set of nucleotide analogue ProTides against ZIKV and obtain basic structure-activity relationships, we selected a set of nucleotides designed to inhibit viral RdRPs by three different mechanisms: obligate chain termination, nonobligate chain termination, and lethal mutagenesis (Figure 1) [40,41]. Past work—and the successful results of drug development against HCV—shows that nucleosides alone lack adequate potency owing to slow phosphorylation in the target cells [42]. McGuigan’s nucleotide aryloxy phosphoramidates include amino acid esters and are well established; sofosbuvir includes this functional group, and we chose it as our starting point [9,43]. One potential drawback of the McGuigan design is that unmasking is initiated by the action of carboxyesterases, which are highly active in the hepatocytes targeted by HCV but less active in the neural cell targets of ZIKV [42]. For that reason, we chose to investigate a recently developed methylthioethyl tryptamine ProTide from Wagner, which is unmasked by chemical cleavage of methylthioethanol followed by tryptamine removal by HINT1 [26]. HINT1 also catalyzes P-N bond cleavage in the classic McGuigan ProTide. Our hypothesis was that the carboxyesterase-independent unmasking of the methylthioethyl tryptamine ProTide would provide greater potency against ZIKV in human neural stem cells.

The selection of nucleoside analogues for testing focused on ribose modifications known to induce chain termination in vitro when used as a nucleoside triphosphate analogue with the viral RdRP. Some of the selected compounds, e.g., the 3′-deoxy and the 3′-*O*-methyl, are obligate chain terminators, but phosphorylation of this class of compounds in cells is often inefficient [18,19]. Better success in antiviral drug development has been found when using nonobligate terminators, especially 2′ modifications that alter the nucleoside conformation and prevent extension after incorporation during RNA synthesis [14,31]. We included sofosbuvir in this set as a point of reference, along with ProTides of the 2′-*C*-methyl ribosides, which have known potency against the Flaviviridae family [19,31,44]. Also included were new ProTides of 2′-*C*-ethynyl- and 2′-*C*-ethenyluridine. The 2′-*C*-ethynyl ribosyl triphosphates are known sub-μM inhibitors of flavivirus RdRPs [31,45], and we prepared the new 2′-*C*-ethenyluridine ProTide to probe the active site of the RdRP and the metabolic consequences of further modification of the 2′-*C-*β position. A 5-fluorouridine ProTide was included in our set of analogues to test inhibition by lethal mutagenesis, although toxicity of mutagenic nucleoside analogues is typically prohibitive of clinical applications.

### 3.2. Activity against ZIKV and Toxicity of Nucleoside Analogue ProTides in Human Neural Stem Cells

To profile the activity and toxicity profiles of our library of ProTides, we screened compounds in a 20-point, two-fold dose response with a highest concentration of 50 µM against ZIKV PRVABC59/Human/2015/Puerto Rico and ZIKV H/PAN/2016/BEI-259634 in neural stem cells (MOI of 10) using a luminescent cell-viability assay (CellTiter Glo) and determined EC_50_ (effective concentration 50) and CC_50_ (cytotoxic concentration 50) values from these data (Table 1). We observed that the compounds with the best selectivity indexes (SIs) were the 2′-*C*-methyluridine aryloxyl phosphoramidate ProTide (EC_50_PRVABC59: 1 µM, EC_50_H/PAN: 2 µM, CC_50_: 47 µM, SIs: >47, >23, respectively) and the 2′-*C*-ethynyluridine aryloxyl phosphoramidate ProTide (EC_50_PRVABC59: 0.3 µM, EC_50_H/PAN: 0.8 µM, CC_50_: >50 µM, SIs: >166, >62, respectively). These outperformed sofosbuvir in terms of anti-ZIKV activity (EC_50_PRVABC59: 35 µM, EC_50_H/PAN: 46 µM, CC_50_: >50 µM, SIs: >1.4, >1.1, respectively). Activity was observed for the 2′-*C*-methyladenosine aryloxyl phosphoramidate ProTide (EC_50_PRVABC59: 10 µM, EC_50_H/PAN: 18 µM, CC_50_: 42 µM, SIs: >4, >2, respectively) and 2′-*C*-methylcytidine aryloxyl phosphoramidate ProTide against PRVABC59 strain (EC_50_PRVABC59: 48 µM, CC_50_PRVABC59: >50 µM, SI: >1), albeit at lower levels. None of the other ProTides tested displayed activity. Interestingly, the 2′-*C*-ethenyluridine aryloxyl phosphoramidate ProTide was completely inactive and quite toxic to the host cells, suggesting that this functional group may be more promiscuous in terms of target binding or reactive with cellular components [46]. Replacement of the classic McGuigan ProTide masking group with methylthioethyl tryptamine phosphoramidate for the 2′-*C*-methyluridine ProTide completely abolished activity. This result was not expected because the classic McGuigan ProTides are unmasked with the involvement of both a carboxyesterase and HINT1, whereas the methylthioethyl tryptamine phosphoramidate requires only HINT1 in addition to spontaneous, chemical steps [26]. Clearly, cell line specificity for ProTide strategies must be considered in the design of nucleotide analogue inhibitors of ZIKV.

### 3.3. Protection from ZIKV-Induced Cytopathic Effect by 2′-*C*-methyluridine Aryloxyl Phosphoramidate ProTide and Sofosbuvir in a ZIKV-Sensitive Glioblastoma Stem Cell Line

**Figure 2:**
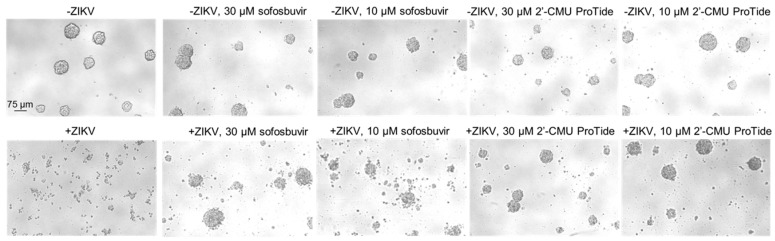
Protection from ZIKV-induced cytopathic effect by 2′-*C*-methyluridine aryloxyl phosphoramidate ProTide and sofosbuvir in a ZIKV-sensitive glioblastoma stem cell model. DMSO-treated GSC 387 cells [-ZIKV and +ZIKV, H/PAN multiplicity of infection (MOI) 10], 10 or 30 µM sofosbuvir-treated cells or 10 or 30 µM 2′-*C*-methyluridine aryloxyl phosphoramidate ProTide (2′-CMU ProTide)-treated cells were incubated for 72 h at 37 °C and 5% CO_2_, and cell foci were subsequently imaged in bright-field.

We and others have previously shown that certain lines of brain tumors with stem cell-like genetic programs, such as glioblastomas, are highly susceptible to ZIKV infection [29,47,48,49]. These cells display a striking phenotypic change upon infection. To highlight the ability of a representative ProTide hit from our library, 2′-*C*-methyluridine aryloxyl phosphoramidate, to protect against ZIKV-induced cytopathic effect, we treated the glioblastoma stem cell line GSC 387 with either 10 or 30 µM 2′-*C*-methyluridine aryloxyl phosphoramidate and compared this to 10 or 30 µM sofosbuvir or the DMSO vehicle control treatment. Samples were prepared in both ZIKV-infected (MOI of 10) and uninfected conditions. After 72 h post-infection, bright-field microscopy images were obtained for each sample and are shown in Figure 2. While both compounds were able to protect against the cytopathic effect at 30 µM (intact cell foci were seen), the 2′-*C*-methyluridine aryloxyl phosphoramidate ProTide was better able to protect against ZIKV-induced cytopathic effect at the lower concentration of 10 µM than sofosbuvir, which is in line with our EC_50_ data in neural stem cells. These data suggest that monosubstituted 2′-*C*-modified uridylate aryloxyl phosphoramidate ProTides may be an attractive chemical series to pursue for more potent and selective ZIKV polymerase inhibitors than sofosbuvir.

### 3.4. Repression of ZIKV Titers by 2′-*C*-methyluridine Aryloxyl Phosphoramidate ProTide and Sofosbuvir in Neural Stem Cells

**Figure 3:**
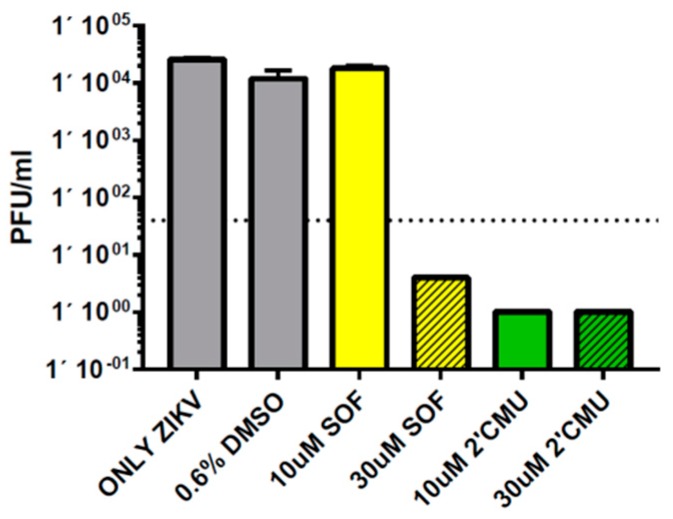
Reduction in ZIKV titers by 2′-*C*-methyluridine aryloxyl phosphoramidate ProTide and sofosbuvir in neural stem cells as observed by plaque assay. Viral infections were conducted in human fetal neural stem cells (H/PAN ZIKV, MOI 0.1) using vehicle-untreated (ONLY ZIKV), DMSO vehicle-treated, 10 or 30 µM sofosbuvir (SOF)-treated, and 10 or 30 µM 2′-*C*-methyluridine aryloxyl phosphoramidate ProTide (2′CMU)-treated samples. The dotted line represents the limit of detection of the assay. Virus was titered as described in the methods section and plaque forming units (PFU) per ml were calculated for each sample, ± standard error (SE), *n* = 4.

To further assess the antiviral activity of the 2′-*C*-methyluridine aryloxyl phosphoramidate ProTide against ZIKV, we performed plaque assays at 48 h post-infection to titer ZIKV (H/PAN, MOI of 0.1) levels during treatment with either 10 or 30 µM of 2′-*C*-methyluridine aryloxyl phosphoramidate ProTide or 10 or 30 µM of sofosbuvir in human fetal neural stem cells (Figure 3). At 30 µM compound, both 2′-*C*-methyluridine aryloxyl phosphoramidate ProTide and sofosbuvir were able to repress viral titers to undetectable levels. However, only 2′-*C*-methyluridine aryloxyl phosphoramidate ProTide was able to reduce ZIKV titers to undetectable levels at the 10 µM compound, again highlighting its superior antiviral activity compared to sofosbuvir.

### 3.5. Enzymatic Incorporation of the Active Triphosphate Metabolites of 2′-*C*-methyluridine and Sofosbuvir Over Time Reveals Higher Levels of Incorporation for the Former Compound

**Figure 4:**
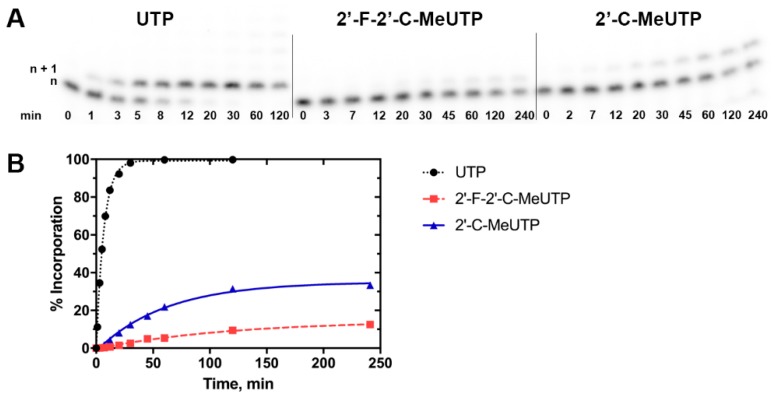
Kinetic characterization of nucleotide and nucleotide analogue incorporation by ZIKV RdRP. (**A**) ZIKV RdRP and primer/template (P/T) substrate (n) was mixed with excess UTP, 2′-F-2′-*C*-methyluridine triphosphate (2′-F-2′-C-MeUTP), or 2-*C*-methyluridine triphosphate (2′-C-MeUTP), and the reaction was quenched at the indicated timepoints. The substrate and the single nucleotide extension product (n + 1) were separated by using a 20% polyacrylamide denaturing gel, and bands were quantified to determine percent incorporation and plotted against time. (**B**) A single exponential fit was used to determine an observed rate of incorporation, *k*_obs_ (% incorporation min^−1^) of 0.151 ± 0.004, 0.0085 ± 0.001, and 0.016 ± 0.001, for UTP (dotted black line), 2′-F-2′-C-MeUTP (dashed red line), and 2′-C-MeUTP (solid blue line), respectively. SE is reported as deviation from the fit, *n* = 2.

Single nucleotide incorporation assays were used to compare the relative preference of ZIKV RdRP for UTP versus the UTP analogs 2′-*C*-methyluridine triphosphate and 2′-fluoro-2′-*C*-methyluridine triphosphate (the active form of sofosbuvir) (Figure 4). Both analogues appeared to serve as chain terminators of ZIKV RdRP polymerization, as previously reported [19,31]. Unsurprisingly, UTP was most efficiently incorporated, with 50% incorporation by 5 min and full incorporation by ~30 min, followed by misincorporating extension. The 2′-fluoro-2′-*C*-methyluridine triphosphate had significantly less efficient incorporation by ZIKV RdRP, as previously reported [19,31,50]. Incorporation plateaued at 15%, and half maximal incorporation was only reached after nearly 90 min. In contrast, 2′-*C*-methyluridine triphosphate reached half of its eventual maximal incorporation of 35% after ~45 min, showing improved incorporation over the active form of sofosbuvir. These trends are supported by previous work focusing on biochemical characterization of 2′-*C*-methyluridine triphosphate and 2′-fluoro-2′-*C*-methyluridine triphosphate incorporation [19,20]. Importantly, here we verify the preference of 2′-*C*-methyluridine over sofosbuvir in both biochemical and cell-based assays, suggesting a robust inhibitor testing pipeline that supports the hypothesis that cellular efficacy occurs as a result of RdRP inhibition by nucleoside analogs.

### 3.6. Molecular Superpositioning of the ZIKV NS5 Active Site: Consequences for 2′-C-Derivatitized Nucleoside Analogues

**Figure 5:**
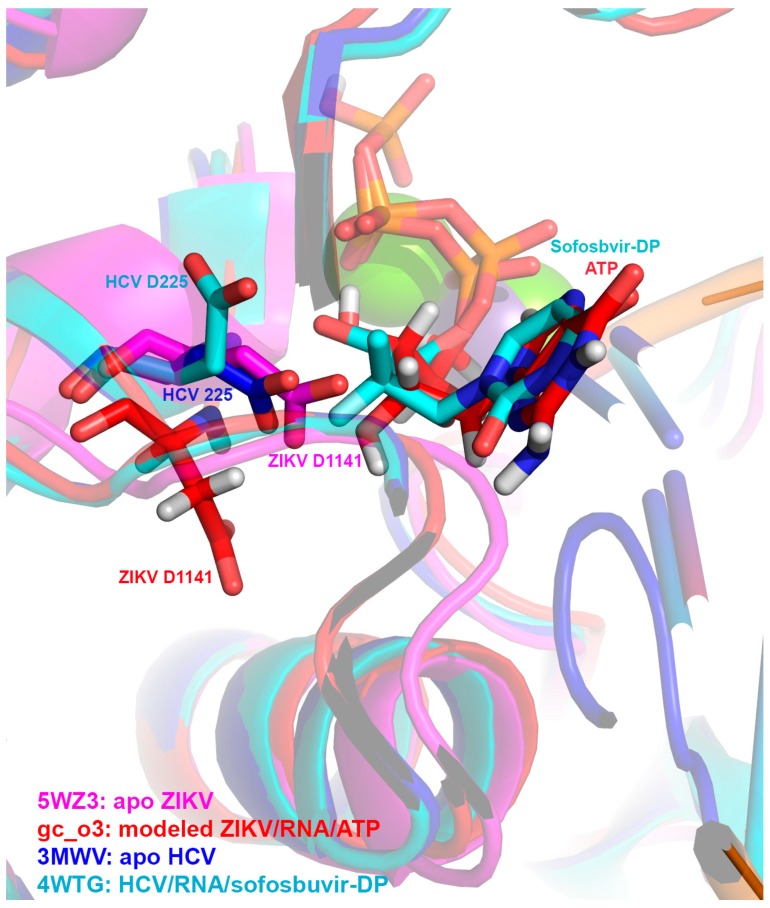
Structural superpositioning of apo and bound ZIKV RdRP and hepatitis C virus (HCV) RdRP. The ZIKV RdRP model gc-o3 [34] in complex with two manganese ions (green), ATP (red/CPK coloring), and RNA (orange/CPK coloring) is shown in red, and a crystal structure of apo ZIKV RdRP is shown in magenta (PDB 5WZ3 [38]). A crystal structure of HCV RdRP complexed with sofosbuvir diphosphate (cyan/CPK coloring), RNA (orange/CPK coloring), and a manganese ion (purple) is shown in cyan (PDB 4WTG [36]), and an apo structure of HCV RdRP is shown in blue (PDB 3MWV [39]). A comparison of the homologous residue D225 (HCV RdRP) and D1141 (ZIKV RdRP) is highlighted in stick form.

A previously prepared model (gc-o3 [36]) of ZIKV RdRP in complex with RNA and ATP generated from PDB 5TFR [37] and PDB 4WTD [34], an apo structure of ZIKV RdRP (PDB 5WZ3 [38]), and an apo structure of HCV RdRP (PDB 3MWV [39]) were aligned with a crystal structure of HCV RdRP complexed with RNA and sofosbuvir diphosphate (PDB 4WTG [34]), shown in Figure 5. ZIKV RdRP is shown in magenta (apo form) or in red (in complex with ligands), and HCV RdRP is shown in dark blue (apo form) or in cyan (in complex). The crystal structure of inhibited HCV RdRP (cyan) shows that residue D225 moved upward to accommodate sofosbuvir diphosphate, while in the ZIKV model (red) [36], the corresponding residue D1141 in ZIKV RdRP pointed down in the opposite direction to accommodate the nucleotide (Figure 5). While the position of the aspartic acid in both the apo forms of the viral RdRPs was similar, it is interesting that the nucleoside ligands were accommodated in different ways, with ZIKV RdRP displaying a larger change in positioning in this residue. While modeling was used to generate the holo-structure of ZIKV RdRP, this suggests some evidence that the ZIKV binding pocket is poised to accommodate a variety of nucleoside analogues. 

## 4. Discussion

In this work, we demonstrated that ProTides other than sofosbuvir have promise as potential antiviral drugs to treat ZIKV infections. Interestingly, an apparent bias towards 2′-*C*-modified uridylate ProTides (with the exception of the ethenyl derivative) was observed, suggesting a possible nucleobase bias to compound activity. Recent biochemical data examining levels of off-target incorporation of nucleoside analogue triphosphates by the human mitochondrial RNA polymerase may help explain the phenotypic relationship we observed; both 2′-*C*-methyluridine triphosphate and 2′-*C*-methyl-2′-*C*-fluorouridine triphosphate (the active metabolite of sofosbuvir) show much lower levels of incorporation by this host enzyme than 2′-*C*-modified analogues with other nucleobases [51]. We propose that this may account for the better safety profile observed for sofosbuvir compared to other 2′-*C*-modified compounds that were tested in clinical trials. This may also suggest that there is greater selectivity for 2′-*C*-modified uridylate incorporation by flavivirus polymerases over host polymerases, though the exact mechanism by which this occurs should be examined in future studies.

We also observed that changing the ProTide functionality of 2′-*C*-methyluridine from an aryloxyl phosphoramidate masking group to a 2-(methylthio)ethyl phosphoramidate resulted in a complete loss of activity, suggesting that the local metabolic environment of the host cell is critical for processing of the ProTide functionality. Further studies examining the relationship between different ProTide functionalities and the cell type dependence of their antiviral activity as well as testing the impact of these effects using in vivo animal model testing are planned in our research groups.

We have demonstrated here for the first time that ProTide technology can be broadly applied to anti-ZIKV drug development with nucleobase and ProTide group selectivity observed. These data will guide future work to design highly selective, safe, and bioavailable compounds for the treatment of ZIKV infections.

## Figures and Tables

**Table 1:** Anti-ZIKV activity and toxicity of nucleoside analogue ProTides in neural stem cells.

Nucleoside Analogue ProTide	EC_50_ PRVABC59 ZIKV, µM	EC_50_ H/PAN ZIKV, µM	CC_50_, µM	SI ^1^ PRVABC59 ZIKV	SI ^1^ H/PAN ZIKV
2′-*C*-Methylcytidine aryloxyl phosphoramidate	48 ± 1	>50	>50	>1	>1
3′-Deoxyadenosine aryloxyl phosphoramidate	>50	>50	>50	>1	>1
5′-Fluorocytidine aryloxyl phosphoramidate	>50	>50	10 ± 1	>0.2	>0.2
3′-*O*-Methyluridine aryloxyl phosphoramidate	>50	>50	>50	>1	>1
2′-*O*-Methylcytidine aryloxyl phosphoramidate	>50	>50	>50	>1	>1
2′-*C*-methyluridinearyloxyl phosphoramidate	1 ± 1	2 ± 1	47 ± 1	>47	>23
2′-*C*-Methyladenosine aryloxyl phosphoramidate	10 ± 1	18 ± 1	42 ± 1	>4	>2
3′-Deoxycytidine aryloxyl phosphoramidate	>50	>50	>50	>1	>1
3′-Deoxy-3′-Fluoroguanosine aryloxyl phosphoramidate	>50	>50	>50	>1	>1
2′-*C*-Ethenyluridine aryloxyl phosphoramidate	>50	>50	10 ± 1	>0.1	>0.1
2′-*C*-Ethynyluridine aryloxyl phosphoramidate	0.3 ± 1	0.8 ± 1	>50	>166	>62
2′-*C*-methyluridine 2-(methylthio)ethyl phosphoramidate	>50	>50	>50	>1	>1
Sofosbuvir	35 ± 1	46 ± 1	>50	>1.4	>1.1

^1^ SI = selectivity index: CC_50_/EC_50_ for a given viral strain. Data values are the represented ± standard error (SE), *n* = 4.
